# IGF-1-Induced Enhancement of *PRNP* Expression Depends on the Negative Regulation of Transcription Factor FOXO3a

**DOI:** 10.1371/journal.pone.0071896

**Published:** 2013-08-14

**Authors:** Ting Liu, Wenjing Yi, Boya Feng, Zheng Zhou, Gengfu Xiao

**Affiliations:** State Key Laboratory of Virology, Wuhan Institute of Virology, Chinese Academy of Sciences, Wuhan, China; Ludwig Maximilians University Munich, Germany

## Abstract

The conformational conversion of the cellular prion protein (PrP^C^) into its β-sheet-rich scrapie isoform (PrP^Sc^) causes fatal prion diseases, which are also called transmissible spongiform encephalopathies (TSEs). Recent studies suggest that the expression of PrP^C^ by the *PRNP* gene is crucial for the development of TSEs. Therefore, the identification of the exogenous and endogenous stimulating factors that regulate *PRNP* expression would help to understand the pathogenesis of TSEs. Here, we demonstrate that forkhead box O3a (FOXO3a) negatively regulates *PRNP* expression by binding to the *PRNP* promoter, which is negatively regulated by insulin-like growth factor 1 (IGF-1). Our results show that the IGF-1-induced enhancement of *PRNP* mRNA and protein levels is due to the activation of the PI3K-Akt signaling pathway. The activation of Akt then induces the phosphorylation of FOXO3a, leading to its translocation from the nucleus to the cytoplasm and preventing its binding to the *PRNP* promoter. Treatment with the PI3K-Akt inhibitor LY294002 induces the nuclear retention of FOXO3a, which leads to a decrease in *PRNP* expression. We present a new IGF-1-PI3K-Akt-FOXO3a pathway, which influences *PRNP* expression. The results of this work are vital for understanding the function of PrP^C^ and for future therapeutic approaches to human TSEs.

## Introduction

The conformational conversion of the cellular prion protein (PrP^C^) into its β-sheet-rich scrapie isoform (PrP^Sc^) causes fatal prion diseases, which are also called transmissible spongiform encephalopathies (TSEs).The TSEs include scrapie, bovine spongiform encephalopathy (BSE), and human Creutzfeld-Jacob disease (CJD) [1], [2]. PrP^C^, which is encoded by the house-keeping gene *PRNP*, is a surface glycoprotein that is expressed in almost all tissues but is expressed mainly in the central nervous system in adults [3], [4], [5]. Although PrP^C^ has been suggested to function as an antioxidant, a signal transducer, and a metal-binding protein, its function is not well understood [6].

Studies have demonstrated that the expression of cellular PrP is essential to the development of TSEs because *Prnp* knock-out mice are resistant to TSEs [7]. In contrast, PrP over-expression leads to increased susceptibility and shortened incubation time [8]. Therefore, individuals who have a higher level of *PRNP* expression might be expected to have shorter incubation periods following iatrogenic exposure to human prions or exposure to BSE. Thus, the up- and down-regulation of human *PRNP* expression appear to be critical for the pathogenesis of human TSEs, such as CJD and fatal familial insomnia (FFI).

In the past decades, several transcription factors have been identified to regulate *PRNP* expression by binding to the *PRNP* promoter or intron 1. For example, YY1 and Hes-1 have been identified as repressors [9], [10], whereas E4BP4, Atox-1, SP1, MTF-1, and HSTF-1 have been identified as activators [9], [10], [11], [12]. Furthermore, some exogenous and endogenous stimulating factors, such as thermal stimuli, Cu^2+^, hyperbaric oxygen, NO, IGF-1, and nerve growth factor (NGF), are believed to play a role in *PRNP* regulation [12], [13], [14], [15], [16], [17], [18].

Among these factors, IGF-1was appealing because it is a critical neurotropic factor in the central nervous system and promotes neuronal growth, survival, differentiation, neuronal cytoskeletal protein expression, and nascent synapse formation [19], [20], [21], [22], [23]. Correspondingly, its receptor, IGF-1R, is abundantly expressed in the brain [24]. However, little is known about the relationship between IGF-1 and *PRNP* expression. We found conflicting results regarding whether IGF-1 affects *PRNP* expression. Lasmezas, C. concluded that IGF-1 treatment increases the level of *PRNP* mRNA in the PC12 cell line [17], whereas Castelnau, P. demonstrated that IGF-1 has no effect on *PRNP* expression in astrocytes [18]. Other researchers reported that IGF-1 protects against prion peptide-induced cell death in neuronal cells [25] and has potential roles in Alzheimer’s disease (AD) [26] and longevity [27]. Therefore, determining whether and how IGF-1 plays a role in *PRNP* expression appears to be a challenging and meaningful aim.

FOXO3a, a member of the O subfamily of the forkhead transcription factors and a critical downstream molecule of the IGF-1/insulin signaling pathway, has a wide range of functions in response to kinds of stimuli in mammals. In response to IGF-1 or insulin, FOXO3a is phosphorylated by PI3K-Akt and excluded from the nucleus, which prevents its binding to the promoters of its target genes. Thus, this cytoplasmic retention impairs the transcriptional regulation function of FOXO3a (reviewed in [28]).

In this article, we confirm that IGF-1 treatment up-regulates *PRNP* expression both at the mRNA and protein levels in SH-SY5Y and HeLa cells. Then, we show that the downstream PI3K-Akt signaling pathway but not the Ras-MAPK pathway plays a critical role in the *PRNP* expression induced by IGF-1. Additionally, we demonstrate that the transcription factor FOXO3a negatively regulates *PRNP* expression by binding to its promoter, which is impaired by IGF-1 treatment. In summary, we identified a new IGF-1-PI3K-Akt-FOXO3a pathway, which regulates *PRNP* expression and is vital for understanding the function of PrP^C^ and for future therapeutic approaches to human TSEs.

## Materials and Methods

### Plasmid Constructs

The plasmid containing the human *PRNP* promoter from −1593 to +134 was a kind of gift from Wolfgang W. Quitschke (Department of Psychiatry and Behavioral Science, State University of New York at Stony Brook, Stony Brook, NY, USA). The full length of the human *PRNP* promoter was then cloned into the pGL3-Basic vector (Promega, E1751) to yield a pGL3-*PRNP* promoter plasmid named pGL3-1593. To construct the FOXO1 and FOXO3a recombinants, pCMV-SPORT6 vectors containing FOXO1 (HC109727) and FOXO3a (HC123573) were purchased from Yrbio. The sequences encoding the FOXO proteins were cloned into the pcDNA3.1^+^ vector using the following primers: 5′-ACTAGCTAGCATGGCCGAGGCGCCTCAGG-3′ and 5′-GCATGTCTAGATCAGCCTGACACCCAGCT-3′ for FOXO1; 5′-CCCAAGCTTACCATGGCAGAGGCACCGGCTT-3′ and 5′-CATGTCTAGATCAGCCTGGCACCCAGCTC-3′ for FOXO3a. The sequence encoding the FOXO3a protein was also cloned into pEGFP-N1 vector using 5′-CCGCTCGAGGCCACCATGGCAGAGGCACCGGCTT-3′ and 5′-CGCGGATCCACGCCTGGCACCCAGCTCTGAG-3′. All recombinants were verified by DNA sequencing.

### Cell Culture, Transfection, and Drug Treatment

SH-SY5Y and HeLa cells were cultured in DMEM (Hyclone) supplemented with 10% fetal bovine serum (FBS) (Gibco) at 37°C and 5% CO_2_. The cells were transfected at 85–90% confluence with Lipofectamine 2000 for the luciferase assays and over-expression experiments according to the manufacturer. To transiently knock-down the expression of the genes of interest, we used siRNAs targeted to FOXO1 (5′-AAGCCCTGGCTCTCACAGCAA-3′ [29] and 5′-GAGCGTGCCCTACTTCAAG-3′ [30]) and FOXO3a (5′-UCACCUUCAGUAAGCAAGCCGUGCA-3′ [31] and 5′-GAGCUCUUGGUGGAUCAUCdTdT-3′ [32]). In addition, siRNA with a random sequence was used as a negative control. When the cells were 30–50% confluence, they were transfected with each siRNA using Lipofectamine 2000, and the knock-down efficiency was assayed by western blotting and real-time PCR. To identify the roles of IGF-1 and PI3K-Akt in *PRNP* expression, 50 or 100 ng/ml IGF-1(AB Biotech, IM051), 25 µM LY294002 (Beyotime, S1737), 10 µM SP600125 (Beyotime, S1876), 25 µM U0126 (Beyotime, S1901), and 1∶1000 dimethylsulfoxide (DMSO) were used, respectively. The cells were treated for the indicated times and harvested for further experiments.

### Western Blotting Analyses

The cells were lysed in RIPA lysis buffer (50 mM Tris, 150 mM NaCl, 1 mM EDTA, 1% NP40, 0.5% sodium deoxycholate, 0.1% SDS) containing multiple protease inhibitors (Beyotime, P0013C). Insoluble materials were removed by centrifugation, and protein concentration was determined by BCA Protein Assay Kit (Beyotime, P0012). Equal amounts of protein were size-fractionated using 12% SDS-PAGE and electro-transferred onto a PVDF membrane (Millipore). Blocked with 5% nonfat milk in TBST (10 mM Tris, 150 mM NaCl, 0.1% Tween-20, pH 7.4) for 1 hour, the membrane was then incubated with specific primary antibodies over night at 4°C (i.e., a 1∶1000 dilution of the anti-PrP monoclonal antibody 8H4 [Abcam, ab61409]; 1∶1000 dilutions of monoclonal antibodies against FOXO1 [Beyotime, AF603], FOXO3a [Cell Signaling Technology, 75D8], phosphorylated FOXO1-Thr-24/FOXO3a-Thr-32 [Beyotime, AF605]; or a 1∶2000 dilution of the anti-actin antibody [Cali-bio, CB100997M]). The blots were then incubated with HRP-conjugated secondary antibody (Proteintech) for 1 hour and detected by a chemiluminescence system (Millipore, WBKL S0500).

### Real-time PCR

Cells treated with siRNA or drug were incubated for the indicated times (72 hours for knock-down experiments) and harvested. Total RNA was isolated using TRIzol reagent (Invitrogen), and an equal amount of RNA was reverse-transcribed using M-MLV Reverse Transcriptase (Promega, M170A) following the manufacturer’s instructions. The reverse transcripts were then analyzed using Fast SYBR Green Master Mix (ABI, 4385612) in a quantitative real time-PCR system (ABI, Stepone). The primers used were as follows: human PrP^C^, forward 5′-AATCAAGCAGCACACGGTCA-3′ and reverse 5′-TCGGTGAAGTTCTCCCCCTT-3′ [33]; human FOXO1, forward 5′-TGGACATGCTCAGCAGACATC-3′ and reverse 5′-TTGGGTCAGGCGGTTCATAC-3′ [34]; human FOXO3a, forward 5′-CCCAGCCTAACCAGGGAAGT-3′ and reverse 5′-AGCGCCCTGGGTTTGG-3′ [35]; and human GAPDH, forward 5′-TGGGCTACACTGAGCACCAG-3′ and reverse 5′-CAGCGTCAAAGGTGGAGGAG-3′ [33]. To quantify the possible variation in the PrP mRNA levels after the treatments, an endogenous reference gene, GAPDH, was set as the control.

### Electrophoretic Mobility Shift Assay (EMSA)

The oligonucleotide sequences of the target probes containing the FOXO binding site are as follows: sense strand of probe 1, 5′-ATGGCCCAGGCACTGTTTACAGCAGCTCT-3′ (−1299 to −1292) and sense strand of probe 2, 5′-TTGATGCACATATGTTTACAATGCAGCCTC-3′(−1177 to −1170). The oligonucleotide used as the cold probe competing for probe 1 and probe 2, which includes the FOXO binding element, is: 5′-ATGGCCCAGGCACTGTTTACAGCAGCTCT-3′. The mutated probe 5′-ATGGCCCAGGCACTGCTGACAGCAGCTCT-3′ was designed with two mutated nucleotides (CTG**T**T**T**AC to CTG**C**T**G**AC). The target and mutant DNAs were labeled with biotin at their 3′-termini. Before use, two complementary oligonucleotides for each probe were annealed to create double-stranded oligonucleotides. HeLa cells were transfected with pcDNA3.1^+^-FOXO3a or pcDNA3.1^+^ for 24 hours and then harvested in phosphate buffered saline (PBS) by centrifugation. The pelleted cells were used for nuclear protein extraction with the Nuclear and Cytoplasmic Protein Extraction Kit (Beyotine, P0027) according to the manufacturer’s protocol. After measurement of the protein concentration using the BCA protein assay, the nuclear protein samples with or without FOXO3a over-expression were ready for EMSA. 1 µM biotin-labeled probes were incubated with 10 µg,15 µg or 20 µg HeLa cell nuclear extracts (NEs) in gel-shift binding buffer (poly[dI-dC], DTT, glycerol, EDTA, NaCl, MgCl_2_, Tris, Beyotime, GS009-1) at room temperature for 20 minutes and then loaded onto a 6% polyacrylamide gel in 0.5×TBE buffer for electrophoresis on ice at 120 V for approximately 1 hour. Then, the binding reaction mixture was transferred onto a positively-charged Nylon membrane (Pierce) and cross-linked using ultraviolet light at 120 mJ/cm^2^ for 60 seconds. The biotin-labeled DNA signals were detected by sequential incubations of the membrane with a streptavidin-HRP conjugate (Beyotime, GS009-6) and the BeyoECL Plus Reagent (Beyotime, GS009-4, GS009-5). To further confirm the specific binding between the target probes and FOXO3a, competition experiments with a 50-fold excess of cold probe and mutation experiments with 1 uM mutated probes were incubated with 10 ug NEs following the same protocol.

### Luciferase Assay

To verify that the recombinant plasmid pGL3-1593 has promoter-like activity, SH-SY5Y and HeLa cells were seeded into 24-well plates and then transfected with 800 ng/well of the pGL3-1593 plasmid as well as the pGL3-Basic and pGL3-Control (Promega, E1751) plasmids as the negative and positive controls, respectively. To determine the influence of FOXO proteins on the *PRNP* promoter, FOXO1 and FOXO3a were each co-transfected with pGL3-1593. The pRL-TK plasmid (Promega, E2241) was set as the internal reference to normalize the transfection efficiency and cell death rate. The cells were harvested at the indicated time points and analyzed for Firefly and Renilla luciferase activities by Dual-Luciferase Reporter Assay Kit (Promega, E1910) and the GloMax Multi Detection System (Promega). .

### Fluorescence Microscopy

To detect the translocation of FOXO3a induced by IGF-1 and signaling pathway inhibitors, HeLa cells were grown to 80–85% confluence in a 12-well plate and transfected with the pEGFP-N1-FOXO3a plasmid using Lipofectamine 2000. After 24 hours, the cells were observed under a fluorescence microscope (Olympus, IX71). Then, cells were treated with 100 ng/ml IGF-1, 25 µM LY294002, 10 µM SP600125, 25 µM U0126, serum starvation, or 1∶1000 DMSO as a control. After 1 hour and 4 hours, the fluorescence was observed to determine the variation with that observed before treatment.

### Statistics

Data were analyzed with GraphPad Prism 5.0 software and all values are given as mean ± standard error of mean (S.E.M.). Student’s *t* test was employed to determine significant differences between the control and experimental test groups. Differences with *p*<0.05 were considered significant.

## Results

### IGF-1 Treatment Increases *PRNP* Expression at both the Protein and mRNA Levels

**Figure 1:**
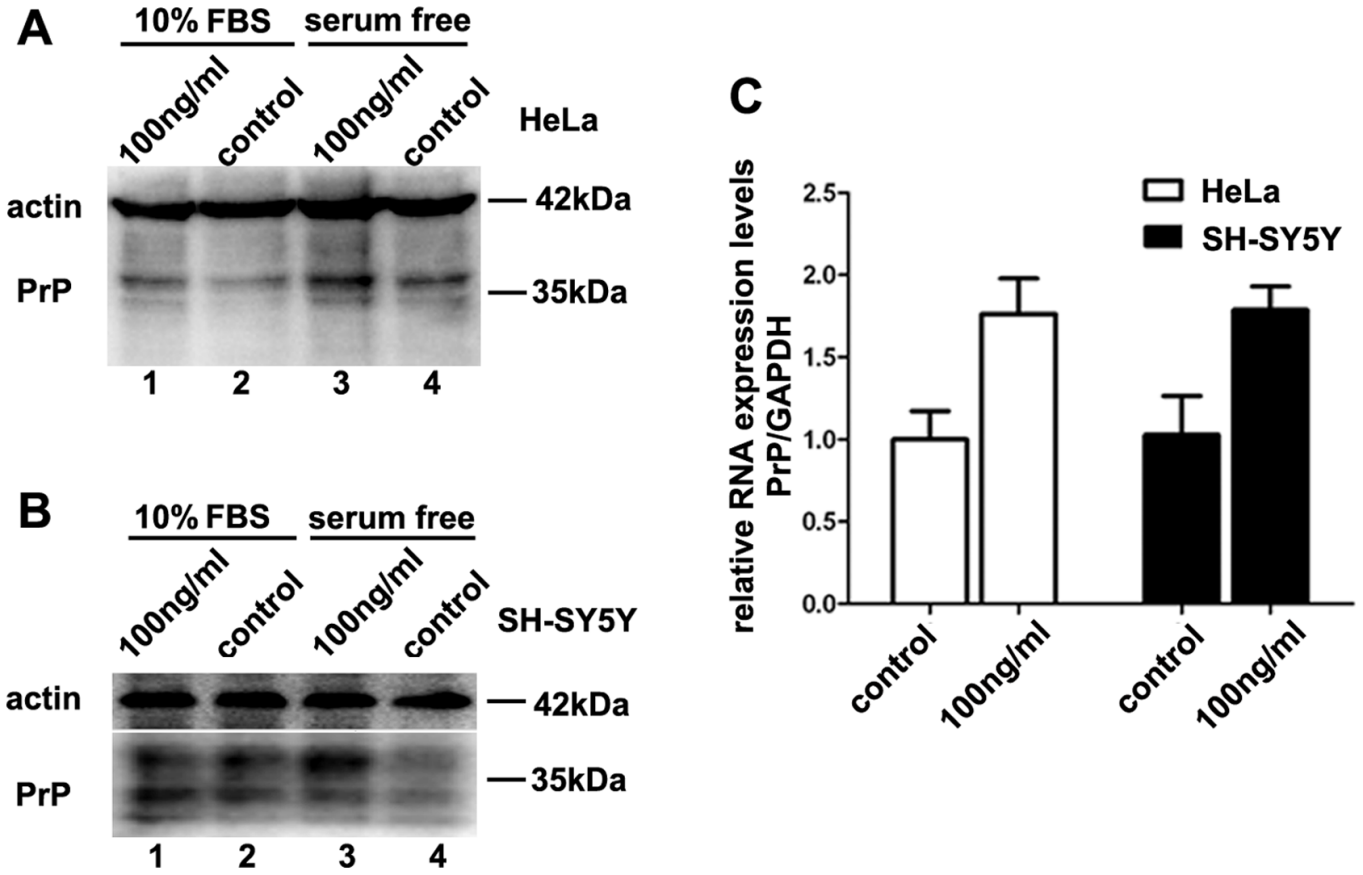
IGF-1 treatment up-regulates *PRNP* expression at both the protein and mRNA levels. (A) and (B) Western blotting analysis for the IGF-1-induced up-regulation of PrP^C^ both in conditions of 10% FBS and serum starvation. Compared with the control group, 100 ng/ml IGF-1 treatment for 24 hours increased the PrP^C^ protein levels in the HeLa and SH-SY5Y cell lines. (C) Quantitative analysis of the IGF-1-induced increase of the *PRNP* mRNA levels in the SH-SY5Y (black columns) and HeLa (white columns) cell lines. The level of *PRNP* mRNA was normalized to that of GAPDH mRNA and expressed as a fold change compared to the mRNA level in the untreated group. All experiments were performed three to five times, and the data were analyzed as the mean ±S.E.M. (*P*<0.05).

To investigate whether IGF-1 has an effect on *PRNP* expression, HeLa cells were seeded into a 12-well plate and cultured with 10% FBS. 24 hours later, cells were treated with 100 ng/ml IGF-1 for another 24 hours. Then, the cells were harvested for western blotting analysis. According to our results, the 100 ng/ml IGF-1 treatment markedly increased the PrP protein level in the HeLa cell line (Fig.1A, lanes 1 and 2). Given that FBS already contains IGF-1, cells were also cultivated in media without FBS and treated with 100 ng/ml IGF-1. In accordance to cells grown in 10% FBS, IGF-1 treated group showed a notable increase of PrP protein level in serum free condition (Fig.1A, lanes 3 and 4). To illustrate that the IGF-1-induced increase in *PRNP* expression is not specific to the HeLa cell line, the neuroblastoma cell line SH-SY5Y was treated with the same conditions, leading to similar results (Fig. 1B). To further identify whether the IGF-1-induced elevation of the PrP protein occurs at the transcriptional level, real-time PCR was conducted to compare the level of PrP mRNA with that of the control GAPDH mRNA in both the HeLa and SH-SY5Y cell lines. The *PRNP* mRNA increased by 1.7±(0.2)-fold after treatment with 100 ng/ml IGF-1 (Fig. 1C). Altogether, these results suggest that IGF-1 up-regulates the expression of *PRNP* at both the protein and mRNA levels.

### IGF-1-induced Enhancement of *PRNP* Expression Depends on the Activation of PI3K-Akt Signaling Pathway

**Figure 2:**
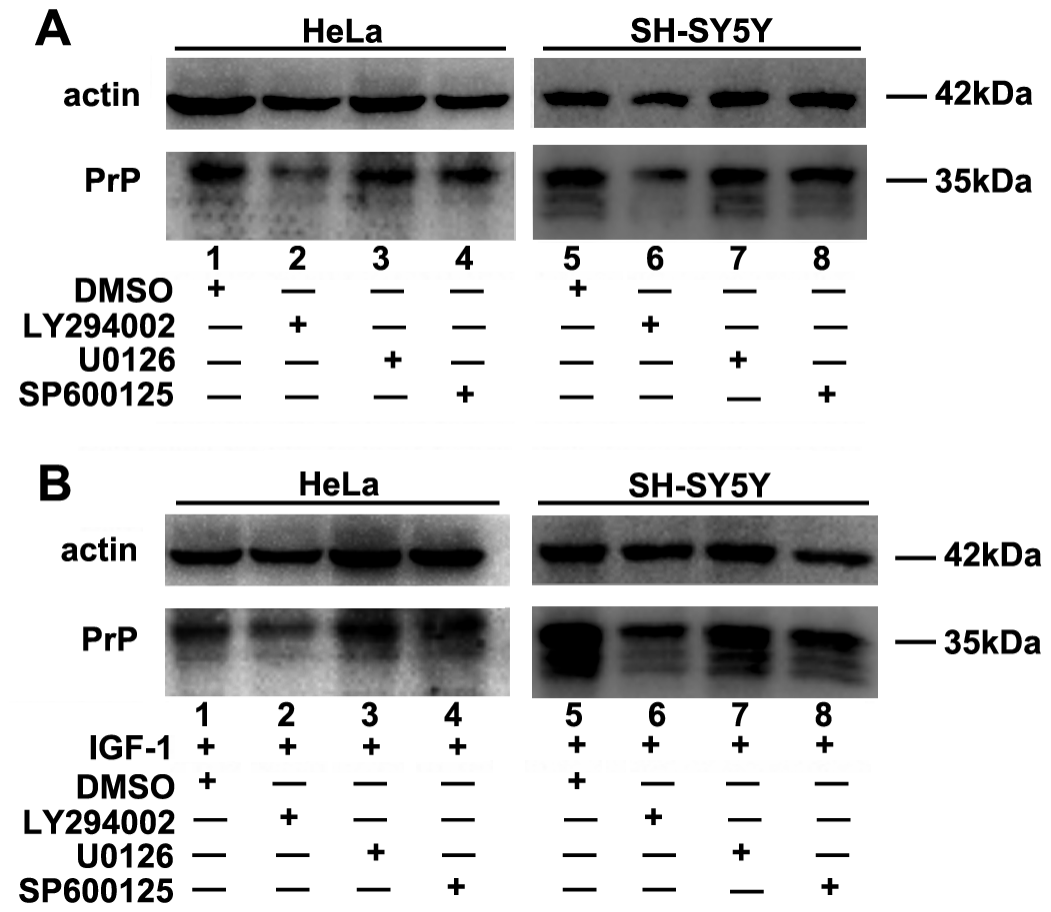
IGF-1-induced enhancement of *PRNP* expression depends on the activation of PI3K-Akt signaling pathway. (A) Variation in the level of *PRNP* expression in HeLa and SH-SY5Y cells treated with different kinase inhibitors. Compared with the control DMSO group (lanes 1 and 5), the PI3K-Akt inhibitor LY294002 treatment group (lanes 2 and 6) demonstrated a notable reduction in the PrP^C^ protein level. In contrast, the MEK1/2 inhibitor U0126 (lanes 3 and 7) and the JNK inhibitor SP600125 (lanes 4 and 8) had little effect on the *PRNP* expression. (B) Similar results were obtained when the HeLa and SH-SY5Y cell lines were co-treated with IGF-1 and inhibitors. The LY294002-treated cells (lanes 2 and 6) showed a remarkable decrease in the PrP^C^ protein level compared with the DMSO-treated group (lanes 1 and 5). In contrast, the U0126-treated groups (lanes 3 and 7) and the SP600125-(lanes 4 and 8) showed no obvious variation. All experiments were performed three times.

IGF-1 initiates signal transduction by binding to its receptor IGF-1R. Once activated by IGF-1, IGF-1R triggers the downstream cascade reactions, the Ras-MAPK pathway and the PI3K-Akt pathway. To clarify which of these two pathways functions in *PRNP* regulation, signaling pathway inhibitors were employed. As shown in Fig.2A, variation in the PrP^C^ protein levels was examined after treatment with the PI3K-Akt inhibitor LY294002 (25 µM), the JNK inhibitor SP600125 (10 µM), the MEK1/2 inhibitor U0126 (25 µM), or DMSO (1∶1000) for 24 hours. Compared with the DMSO control group (lanes 1 and 5), the LY294002-treated group (lanes 2 and 6) demonstrated a notable decrease in the PrP^C^ protein level, whereas the U0126-treated groups (lanes 3 and 7) and SP600125-(lanes 4 and 8) showed no remarkable difference. To investigate whether LY294002 treatment would inhibit the increase of PrP protein level triggered by IGF-1, cells were co-treated with IGF-1 and inhibitors. Further study demonstrated that striking decrease of PrP protein levels were obtained in the condition of LY294002 and IGF-1 co-treatment, whereas little change came out after co-treated with IGF-1 and U0126 or SP600125 (Fig.2B). Interestingly, similar results were achieved both in the SH-SY5Y and HeLa cell lines. Therefore, we surmise that IGF-1 up-regulates *PRNP* expression through the activation of the PI3K-Akt signaling pathway but not the Ras-MAPK signaling pathway.

### FOXO3a Down-regulates *PRNP* Expression at both the mRNA and Protein Levels

**Figure 3:**
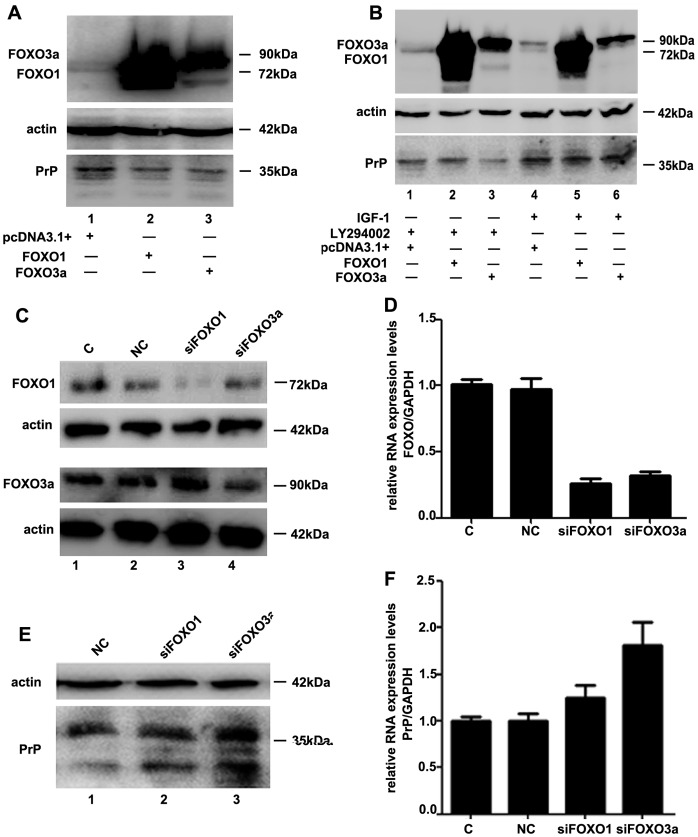
FOXO3a negatively regulates *PRNP* expression at both the mRNA and protein levels. (A) Over-expression of FOXO1 (lane 2) and FOXO3a (lane 3), as detected by western blotting, induced a reduction of PrP protein levels in HeLa cells. (B) Western blotting showing that IGF-1 and LY294002 have an opposite function on the decrease of PrP protein levels induced by FOXO proteins over-expression. LY294002 treatment enhanced the reduction of PrP protein levels triggered by FOXO proteins, especially FOXO3a (lanes 1, 2 and 3). However, 100 ng/ml IGF-1 treatment impaired the negative regulation of FOXO proteins on *PRNP* (lanes 4, 5 and 6). (C) and (D) Western blotting and real-time PCR showing the efficiency of siFOXO1 and siFOXO3a in HeLa cells. Compared with the negative control groups, FOXO1 and FOXO3a were efficiently knocked-down. All experiments were performed at least three times, and the data were analyzed as the mean ± S.E.M. (*P*<0.05). (E) and (F) Western blotting and real-time quantitative PCR showing that the knock-down of FOXO1 and FOXO3a by targeted siRNAs affects *PRNP* expression at both the protein and mRNA levels. Compared with the negative control group, the knock-down of FOXO3a increased the PrP^C^ protein level remarkably, after normalization to actin, whereas the knock-down of FOXO1 had only a slight effect on the PrP^C^ protein level. A similar result was obtained at the mRNA level. All experiments were performed at least three times, and the data were analyzed as the mean ± S.E.M. (*P*<0.05).

Although several transcription factors, such as YY1, Atox1, E4BP4, and p53, have been verified to regulate *PRNP* expression by binding to the *PRNP* promoter, whether the FOXO transcription factors playing a role in *PRNP* expression has not yet been reported. As transcription factors, the FOXO proteins play a critical role in the IGF-1/insulin signaling pathway. Thus, we hypothesized that the FOXO proteins may regulate *PRNP* expression. To verify our hypothesis, the plasmids pcDNA3.1^+^-FOXO1 and pcDNA3.1^+^-FOXO3a were transfected into the HeLa cell line. As shown in Fig.3A, 24 hours after transfection, dramatic increase of FOXO1 (lane 2) and FOXO3a (lane 3) were detected in the over-expression groups at 72kDa and 90kDa, respectively. Compared with the pcDNA3.1^+^ group, the over-expression of FOXO1 and FOXO3a led to a reduction of PrP^C^ protein level (Fig.3A, lanes 2 and 3). As FOXO proteins are critical downstream molecular of IGF-1/insulin signaling pathway, we further investigated into whether IGF-1 and LY294002 could affect the function of FOXO1 and FOXO3a on *PRNP* regulation. As shown in Fig.3B, 100 ng/ml IGF-1 treatment impaired the reduction of PrP protein levels induced by FOXO1 and FOXO3a over-expression (Fig.3B, lanes 4, 5 and 6). In contrast to it, 25 µM LY294002 treatment enhanced the reduction of PrP protein levels triggered by FOXO proteins over-expression (Fig.3B, lanes 1, 2 and 3). To further examine whether the FOXO proteins play a role in *PRNP* expression, we designed siRNAs targeted to FOXO1 and FOXO3a. Western blotting assay and real-time PCR showed that FOXO1 and FOXO3a were efficiently knocked-down by their targeted siRNAs (Fig.3C, lanes 3 and 4; Fig.3D). Compared with the negative controls, the siRNA-induced knock-down of FOXO1 led to a slight increase in the PrP^C^ protein and mRNA levels (Fig.3E, lane 2; and Fig.3F). In contrast, the knock-down of FOXO3a by its targeted siRNAs demonstrated an enhancement in the expression of *PRNP* up to 1.8(±0.3)-fold (Fig.3E, lane 3; and Fig.3F). These results indicate that over-expression of the FOXO transcription factors down-regulates PrP^C^ expression, whereas knock-down of the FOXO transcription factors increases the PrP^C^ expression. Our results indicate that the FOXO transcription factors, especially FOXO3a, have an important role in the regulation of *PRNP* expression.

### FOXO3a Negatively Regulates *PRNP* Expression by Binding to the *PRNP* Promoter

**Figure 4:**
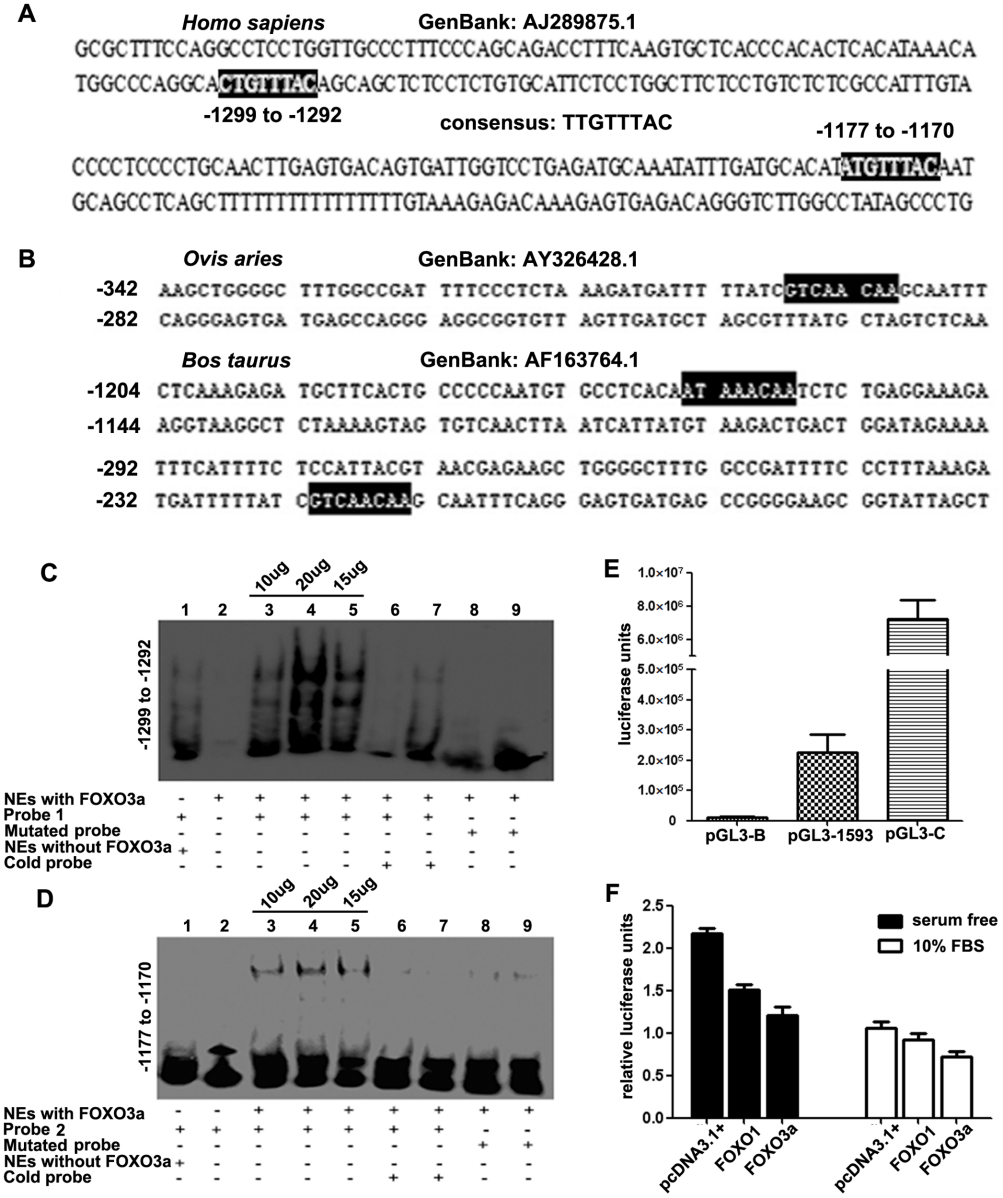
FOXO3a down-regulates *PRNP* expression by binding to its promoter. (A) Two potential FOXO3a binding sites in the human *PRNP* promoter (dark background) at −1299 to −1292 and −1177 to −1170. (B) Potential FOXO3a binding sites in the *Ovis aries* and *Bos taurus PRNP* promoters (dark background). (C) and (D) EMSA showing that FOXO3a can bind to the human *PRNP* promoter. Compared with the control (lanes 1 and 2), FOXO3a bound to the *PRNP* promoter in a dose-dependent manner (lanes 3, 4 and 5), and this binding was abolished by a 50-fold excess of unlabeled probes (lanes 6 and 7) and mutation of the FOXO binding site (lanes 8 and 9) results in low affinity for FOXO3a. (E) Luciferase assay confirming that the human *PRNP* promoter recombinant has promoter-like activity. The pGL3-Basic and pGL3-Control plasmids were used as negative and positive controls, respectively. (F) Luciferase assay showing that FOXO1 and FOXO3a reduce the promoter activity of the *PRNP* promoter in the HeLa cell line. Compared with the control, the co-transfection with FOXO1 and FOXO3a led to a 10–30% decrease in luciferase units in 10% FBS medium, whereas a 30–45% decrease was detected when the cells were serum starved. The ‘relative luciferase unites’ stands for the ratio of firefly/renilla. All experiments were performed three to five times, and the data were analyzed as the mean ± S.E.M. (P<0.05).

As the transcription factor FOXO3a was proven to negatively regulate *PRNP* expression, we next examined whether the FOXO3a-induced down-regulation of *PRNP* expression depends on its binding to the *PRNP* promoter. We scanned the *PRNP* promoter from −1593 to +134 (GenBank AJ289875.1) for putative FOXO3a binding sites (TESS, http://www.cbil.upenn.edu/cgi-bin/tess/tess), and two putative FOXO3a binding sites were found. Relative to the start site, the first is located at −1299 to −1292 (5′-CTGTTTAC-3′), and the second is located at −1177 to −1170 (5′-ATGTTTAC-3′) (Fig.4A). In addition, potential FOXO binding sites were located in the *PRNP* promoters of *Ovis aries* (GenBank AY326428.1) and *Bos taurus* (GenBank AF163764.1) (Fig.4B, dark background). To determine whether FOXO3a could bind to the *PRNP* promoter at the putative sites, we performed EMSAs with biotin-labeled oligonucleotides. Our results showed that FOXO3a could bind to both potential FOXO transcription factor binding sites in the human *PRNP* promoter in a dose-dependent manner (Fig.4C and Fig.4D, lanes 3, 4, and 5) and that this binding could be abolished by competition with a 50-fold excess of unlabeled probe (Fig.4C and Fig.4D, lanes 6 and 7). In contrast, no obvious binding was detected using NEs without FOXO3a over-expression or unlabeled probes (Fig.4C and Fig.4D, lanes 1 and 2). Additionally, a probe with two mutations in the FOXO binding sequences showed low affinity for FOXO3a (Fig.4C and Fig.4D, lanes 8 and 9). These results suggest that the FOXO3a is able to bind to the *PRNP* promoter to regulate *PRNP* expression.

To further investigate whether FOXO3a down-regulates *PRNP* by binding to the *PRNP* promoter, the full-length *PRNP* promoter (−1593 to +134) was cloned into the pGL3-Basic vector. Then, the recombinant plasmid, the negative control pGL3-Basic, and the positive control pGL3-Control were respectively transfected into the HeLa cell line, and transfection with the recombinant plasmid resulted in promoter activity (Fig.4E). The similar results were obtained in SH-SY5Y cell line (data not shown). In addition, co-transfection with plasmids for FOXO1 and FOXO3a expression led to a 10–30% reduction in the *PRNP* promoter activity in the HeLa cell line (Fig.4F, white columns). Compared with it, a more remarkable decrease (30–45%) was detected under serum starvation (Fig.4F, black columns). All of these results prove that transcription factor FOXO3a can bind to the *PRNP* promoter to repress *PRNP* expression.

### The IGF-1-induced Enhancement of *PRNP* Expression Occurs via the PI3K-Akt-FOXO3a Pathway

**Figure 5:**
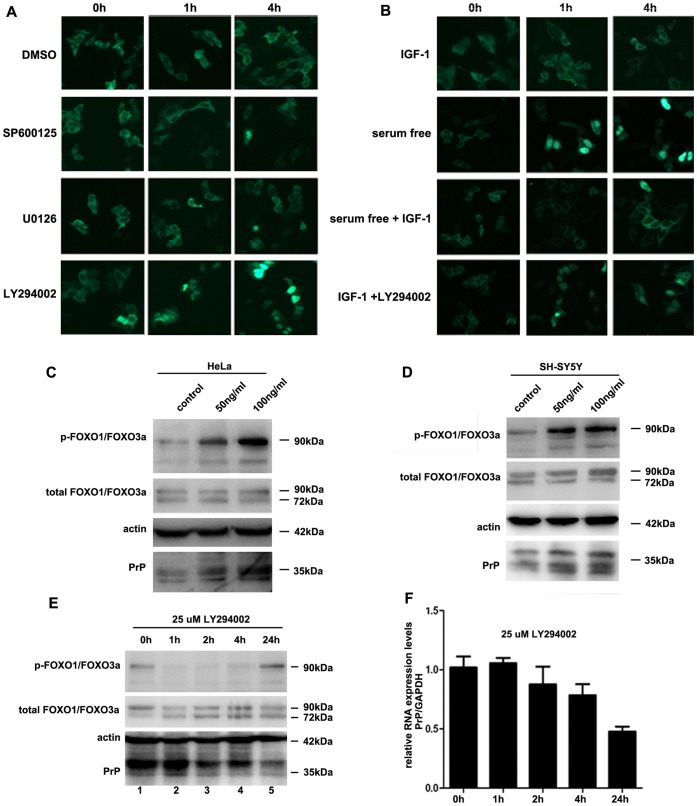
IGF-1 induces an increase in *PRNP* expression through the PI3K-Akt-FOXO3a pathway. (A) and (B) Translocation of FOXO3a induced by different treatments. Before the treatments, GFP-FOXO3a was located predominantly in the cytoplasm. In contrast, serum starvation or treatment with LY294002 induced the nuclear translocation of GFP-FOXO3a, whereas treatment with SP600125 or U0126 had no effect on the GFP-FOXO3a translocation. However, IGF-1 was able to induce the cytoplasmic retention of GFP-FOXO3a even under serum-free conditions, whereas treatment with LY294002 led to the nuclear retention of GFP-FOXO3a even in the presence of IGF-1. (C) and (D) Western blotting showing the relationship between the phosphorylation of FOXO3a and *PRNP* expression. Treatment with 50 and 100 ng/ml IGF-1 remarkably increased the phosphorylation of FOXO3a in both the SH-SY5Y and HeLa cell lines but caused little variation in the total level of FOXO3a. In accordance with this increase in the phosphorylation of FOXO3a, the PrP^C^ protein level was also increased. All protein levels were normalized to actin. (E) Western blotting showing the relationship between the dephosphorylation of FOXO3a and *PRNP* expression. The phosphorylation of FOXO3a was maximally inhibited after treatment with LY294002 for 1 hour, and this inhibition was gradually reduced after 4 hours. In addition, the PrP^c^ protein levels began to decrease after treatment with LY294002 for 2 hours, with the maximal decrease appearing after 24 hours. (F) Quantitative PCR showing the variation in the *PRNP* mRNA levels after treatment with LY294002 for different times. No notable changes were detected after 1 hour of treatment, but the *PRNP* mRNA level gradually decreased over time, and an approximately 50% decrease was observed after 24 hours of treatment. All experiments were performed three times, and the data were analyzed as the mean ± S.E.M. (*P*<0.05).

Because we proved that FOXO3a negatively regulates *PRNP* expression by binding to its promoter, we next wanted to determine how this transcription factor participates in *PRNP* regulation in response to IGF-1. First, we confirmed that IGF-1 and inhibitors of PI3K-Akt could induce the translocation of FOXO3a between cytoplasm and nucleus. After 24 hours in DMEM with 10% FBS, the cells were treated with the indicated doses of DMSO, LY294002, U0126, or SP600125 or were subjected to serum starvation. As shown in Fig.5A and Fig.5B, FOXO3a was mainly distributed in the cytoplasm before the treatments. However, after 1 hour of treatment with the PI3K-Akt inhibitor LY294002 and after serum starvation, FOXO3a was relocated to the nucleus. This new localization could be detected after 4 hours of treatment and possibly longer. In contrast, no variation was detected after treatment with either U0126 or SP600125. However, IGF-1 was able to induce the cytoplasmic localization of FOXO3a even under serum-free conditions after 1 and 4 hours of treatment, and this induction could be inhibited by LY294002. These results indicate that IGF-1 treatment activates the PI3K-Akt signaling pathway and that activated Akt then phosphorylated FOXO3a, leading to its cytoplasmic localization. Thus, we next investigated the relationship between FOXO3a phosphorylation and *PRNP* expression. As shown in Fig.5C, the amount of phosphorylated FOXO3a noticeably increased, whereas the total amount of FOXO3a remained almost the same after treatment with 50 or 100 ng/ml IGF-1 for 24 hours in HeLa cells. Meanwhile, the PrP protein levels were up-regulated along with the phosphorylation of FOXO3a. The same results were also observed in the SH-SY5Y cell line (Fig.5D). Then, we hypothesized that the dephosphorylation of FOXO3a would decrease the expression of *PRNP*. According to our results (Fig. 5E), treatment with LY294002 for 1 or 2 hours showed the greatest inhibition of FOXO3a phosphorylation, and this inhibition started to weaken after 4 hours. As expected, the PrP protein level varied little after 1 hour of treatment with LY294002, but began to decrease after 2 hours of treatment, and was lowest after 24 hours. The decrease in the *PRNP* expression upon LY294002 treatment for different times was further confirmed at the mRNA level by quantitative PCR (Fig.5F). The level of *PRNP* mRNA was gradually reduced after LY294002 treatment for 2 hours and approximately 50% decrease was observed after 24 hours. Taken together, these results suggest that IGF-1 treatment activates the PI3K-Akt pathway, which then phosphorylates FOXO3a. The phosphorylation of FOXO3a induces its translocation from the nucleus to the cytoplasm and impairs its transcriptional regulation function by preventing its binding to the *PRNP* promoter, subsequently resulting in the enhancement of *PRNP* expression.

## Discussion

IGF-1 and insulin are critical growth factors that modulate metabolism, growth, cell differentiation, and survival in most mammalian tissues, including the central nervous system, and are mediated by their respective receptors [22], [24], [36], [37]. However, whether IGF-1 plays a role in *PRNP* expression is not well understood. Although conflicting results have been published by Lasmezas, C. and Castelnau, P. [17], [18], our results confirm that 100 ng/ml IGF-1 treatment does increase the PrP protein and mRNA levels in the SH-SY5Y and HeLa cell lines, both of which are derived from *Homo sapiens*. The reasons for these contrasting observations may be related to differences in the cell types and assay systems.

We also present evidence regarding the roles played by the FOXO transcription factors in *PRNP* expression. We found that FOXO3a is essential for *PRNP* expression in response to IGF-1 treatment. According to our results, the over-expression of both FOXO1 and FOXO3a decreased the PrP^C^ protein levels and the activity of the *PRNP* promoter. The knock-down of endogenous FOXO3a enhanced the expression of PrP^C^ at the mRNA and protein levels up to 1.8(±0.3)-fold, whereas the knock-down of FOXO1 showed a little increase. We further demonstrated that FOXO3a could bind to the *PRNP* promoter at two potential sites, from −1299 to −1292 and from −1177 to −1170 upstream of the transcriptional start site. The binding of FOXO3a to the *PRNP* promoter was impaired by IGF-1, which induced the phosphorylation of FOXO3a through the PI3K-Akt signaling pathway and thus excluded it from the nucleus. The inhibition of PI3K-Akt by LY294002 enhanced the binding of FOXO3a to the *PRNP* promoter, which then down-regulated the expression of *PRNP* by inducing the nuclear retention of FOXO3a.

There are four FOXO family members in mammals: FOXO1 (FKHR), which is predominantly expressed in adipose tissue; FOXO3a (FKHRL1), which is predominantly expressed in the heart, brain, kidneys, and ovaries; FOXO4 (AFX), which is predominantly expressed in muscle and heart tissue; and FOXO6, which is predominantly expressed in the brain [38], [39], [40]. All of the FOXO proteins regulate their target genes by binding to their promoters at the consensus binding site TTGTTTAC [41]. The different tissue distributions suggest that each FOXO homolog may play an important, tissue-specific role. In adults, *PRNP* is mainly expressed in the central nervous system, a similar distribution to those of FOXO3a and IGF-1R. Thus, we draw the conclusion that FOXO3a plays a major role in *PRNP* expression due to the dramatic increase of PrP protein level induced by the knock-down of FOXO3a.

The function of the FOXO transcription factors is mainly regulated by post-translational modifications (PTMs), including phosphorylation, acetylation, and mono- and poly-ubiquitination (reviewed in [28]). In addition to phosphorylated by IGF-1/insulin signaling pathway, FOXO proteins are regulated by MST1, JNK, and other kinases in response to oxidative stress. Oxidative stress occurs when cellular antioxidants are decreased, when oxygen radicals are increasingly generated, or when cells are exposed to an extracellular ROS, such as H_2_O_2_ [42]. An increase in intracellular oxidative stress is responsible for the onset and progression of human diseases, including ischemic heart disease, neurodegenerative diseases, such as AD and Parkinson’s disease (PD), and diabetes [43], [44]. The FOXO proteins have a critical role in removing ROS by activating their target genes, such as MnSOD, catalase, and peroxiredoxins [31], [45], [46]. For example, the activation of FOXO4a may play a feedback role to reduce the oxidative stress level by activating MnSOD and catalase [47]. FOXO3a is also believed to regulate the expression of peroxiredoxin II and peroxiredoxin III by binding to their promoters in response to oxidative stress [31], [46]. Recent studies have proven that oxidative stress mimicked by Cu^2+^, hyperbaric oxygen, or hypoglycemia enhances *PRNP* expression [13], [14], [48] and that the lack of PrP^C^ increases the level of intracellular oxidative stress [49], [50], [51], [52]. Thus, whether the FOXO proteins play a role in *PRNP* expression in response to oxidative stress is worthy of attention. As the PTMs of the FOXO proteins are very complex, we only focused on the influence of PI3K-Akt-FOXO3a on the expression of *PRNP* induced by IGF-1. Whether other kinases and signaling pathways regulate *PRNP* expression in response to other stimuli by regulating the FOXO proteins remains unknown. Efforts on this issue may further reveal the function of the FOXO proteins and PrP^C^ and better elucidate the relationship between oxidative stress and prion diseases.

FOXO proteins can function both as transcriptional activators and repressors, likely depending on the co-factors that they recruit upon DNA binding (reviewed in [53], [54]). The first pivotal co-factor is p300/CBP [55], [56], which interacts with the FOXO proteins and acetylates the histidine of the FOXO proteins at different sites. β-Catenin, another co-factor, conservatively interacts with the FOXOs and enhances their transcriptional activity in many species, from *C*. *elegans* to mammals, in response to oxidative stress [57]. Sirt1, a class III histone deacetylase, has an antagonistic role on FOXO activity. Sirt1 increases the expression of genes regulating cell cycle arrest and stress resistance but inhibits the action of FOXOs on apoptotic genes [57], [58], [59]. The current study is the first to describe that FOXO3a plays a negative role in *PRNP* expression by binding to its promoter. However, whether any other co-factors, such as p300/CBP and β-catenin, also affect *PRNP* expression by binding to FOXO3a is unknown.

**Figure 6:**
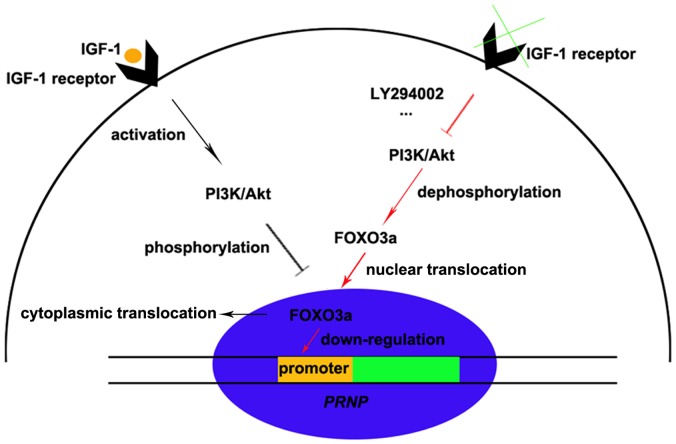
Model for the regulation of *PRNP* in response to IGF-1. After cells are exposed to IGF-1, IGF-1R is activated, which then activates the PI3K-Akt pathway. The activated Akt then phosphorylates FOXO3a, leading to its translocation from the nucleus to the cytoplasm and thereby preventing FOXO3a from binding to the *PRNP* promoter (black arrows and streaks). With no or low levels of IGF-1 or in the presence of LY294002, the negative regulation of PI3K-Akt on FOXO3a is impaired. Then, FOXO3a is able to relocate to the nucleus, where it binds to the *PRNP* promoter and negatively regulates *PRNP* expression (red arrows and streaks).

Based on all of our results, we propose a model to describe how the PI3K-Akt-FOXO3a signaling pathway plays a role in *PRNP* expression in response to IGF-1 (Fig.6). In this model, after cells are exposed to IGF-1, IGF-1R is activated, which then activates the PI3K-Akt pathway. The activated Akt in turn phosphorylates FOXO3a, inducing the cytoplasmic translocation and inhibiting the transcriptional activity of FOXO3a. Subsequently, the binding of FOXO3a to the *PRNP* promoter is prevented, leading to the enhancement of *PRNP* mRNA and protein expression (black arrows and streaks). However, the action of FOXO3a on *PRNP* expression is reversed in the presence of no or low levels of IGF-1 as well as in the presence of the PI3K-Akt inhibitor LY294002. The inactivation of PI3K-Akt results in a low level of phosphorylation and the nuclear retention of FOXO3a. Then, FOXO3a binds to the *PRNP* promoter by itself or possibly with other co-factors, resulting in the down-regulation of the *PRNP* gene (red arrows and streaks). As PrP^C^ has been suggested to play a wide range of roles with different physiological activities and an essential role in the pathogenesis of TSEs, our findings are vital for the understanding of the function of PrP^C^ and will benefit future therapeutic approaches to human TSEs.
